# Which disadvantaged students study medicine? Analysis of an English outreach scheme

**DOI:** 10.1002/hsr2.264

**Published:** 2021-05-06

**Authors:** Carolyn Murray, Anna Mountford‐Zimdars, Karen Mattick

**Affiliations:** ^1^ University of Exeter Medical School Exeter UK; ^2^ Graduate School of Education University of Exeter Exeter UK

**Keywords:** ethics, health policy, medical education, statistics and research methods

## Abstract

**Background:**

Students from disadvantaged backgrounds continue to be underrepresented in medical education. Widening participation (WP) or outreach schemes seek to increase diversity. Drawing on previously unexplored data from a scheme called Realising Opportunities in England, this study aimed to investigate which high‐achieving socioeconomically disadvantaged students in a national WP scheme went on to study medicine at university.

**Methods:**

This retrospective longitudinal study analyzed data of 2665 16‐year‐olds on a WP scheme in England between 2010 and 2014. Descriptive statistics and logistic regression analyses investigated any differences between those that went on to study medicine and those that did not. Eligibility for studying medicine, student's neighborhood, gender, ethnicity, parent's higher education experience, exam attainment, interest in medicine, and their subject of choice for university at age 16 were considered.

**Results:**

Of the 1850 students who were tracked to a university destination, only 55 (3%) studied medicine. Participants with high exam results, female, Asian, and from neighborhoods of higher university entry were most likely to study medicine. In the multivariate model, only prior attainment and intention to study medicine predicted studying medicine. Three hundred and forty Realising Opportunities participants expressed interest in studying medicine at age 16, but 80 (24%) were found to have unrealistic aspirations based on their prior academic attainment.

**Conclusions:**

Attainment and intention were key factors for predicting medicine enrolment among these outreach scheme participants. Some students interested in studying medicine had insufficient academic attainment to compete for medical school places. Efforts to equalize attainment and provide guidance for career choice are crucial factors for students from disadvantaged backgrounds.

## 1 INTRODUCTION

Medical schools worldwide need to increase the number of students they select from socially disadvantaged groups,^1^ with medicine remaining “one of the most inaccessible professions” in the United Kingdom.^2^ However, efforts to reverse the trend of the socioeconomically deprived being underrepresented in medicine have shown minimal success.^3^ This inequality challenges discourses of equality of opportunity^4^ and potentially aggravates doctor recruitment challenges in specific geographic areas. Individuals recruited from underserved areas to study medicine are more likely to return to serve the communities they know.^5^, ^6^, ^7^ A UK study showed that family doctors whose parents had semi‐routine, or routine occupations, were 4.3 times more likely to work in a deprived practice than those with parents from managerial and professional occupations.^5^ Since 2002, the Australian government made it mandatory for 25% of medical students to be from rural backgrounds to address coverage issues,^8^ although the impact has yet to be evaluated.^9^

Encouraging underrepresented socioeconomic groups into higher education has been defined as “widening participation” (WP)^10^, ^11^ in the United Kingdom, or outreach or “diversity and inclusion” in the United States. WP schemes aim to “remove barriers, raise aspirations and improve access to education” for WP students.^12^ Such schemes target participants to consider medicine as a career^5^ by addressing the barriers to application so that medicine is seen as a realistic option.^8^ These barriers include considering medicine as “culturally alien”,^12^ for “posh kids” only^12^; students not perceiving themselves as “doctor material”^13^; it being a choice outside the norm for their peer group^8^; teachers and schools being discouraging in their choice^8^; no one else in their family having studied at higher education^13^; concerns about the selection process or being poorly informed about it^5^, ^13^, ^14^; worries about work‐life balance^13^; the difficulty and length of the course.^13^ A survey of 6492 Australian students found predictors for aspiring to medical careers were high cultural capital, conscious career considerations, speaking English as an additional language, female, and perceiving themselves as “well above average” academically.^15^

Despite understanding these barriers, there is still a lack of WP applicants applying to medical schools: research from a UK medical school found that 80% of applicants came from 20% of schools and these were mainly selective schools or large sixth form colleges.^16^ In 2015, the UK government set the target of “doubling the proportion of pupils from disadvantaged backgrounds going into higher education from 2009 levels.”^16^ However, little progress has been made to achieve this^12^ and the challenge of WP remains a key priority for the medical profession, the government, and universities. There is paucity of research into understanding the factors that have enabled some WP students to be successful in their desire to study medicine. If these could be elicited, the number of WP students in medicine could increase.

Realising Opportunities is an English widening participation program, which provides students with a supported entry route for all university subject areas, and not specifically medicine. This study draws on data from the scheme to discover which high‐achieving socioeconomically disadvantaged students went on to study medicine at university with the aim of discovering factors that could inform recruitment of students from WP backgrounds.

## 2 METHODS

### 2.1 Study design

This study is a longitudinal retrospective analysis of routinely collected data from “Realising Opportunities” (RO),^17^ a nationwide, English widening participation scheme for 16‐ and 17‐year‐olds from WP backgrounds. Participants, to be eligible for the scheme, attended one of the 339 state‐funded English schools that met some marker of school‐level disadvantage (either regarding attainment profile or high percentage of children eligible for free school meals). All participants had achieved good grades in exams at age 16 (at least five excellent passes, ie, A*‐B grades at General Certificate in Secondary Education [GCSE]) and met at least two other markers such as coming from a home where neither parent had attended university and/or living in an area where few people attend university.^17^

Data access was through the RO partnership, within their data protection and legal compliance parameters. Key considerations were that participants had explicitly opted for their data to be used for research purposes and that data were anonymized. Ethical approval was from the University of Exeter.

### 2.2 Data analysis

Realising opportunities participant's data were analyzed from 2010 to 2014, with the exception of year 2011 as consent from participants had not been obtained. RO data on participants were linked to advance level exams at age 18, higher education destination, and university course through the Higher Education Access Tracker (HEAT), which draws information from databases including the Higher Education Statistics Agency (HESA).^18^, ^19^ All data reporting had to conform to HESA requirements to anonymize statistics.^20^

The data set included 2670 participants. All those participants (817), without a university destination, were included in the data when looking at the sociodemographic distribution of the sample but removed when analyzing the participant's Higher Education destination.

We do have a significant amount of missing data in the analysis. This should be considered our first finding in itself: It is surprising for an outreach scheme of the scale and intent as RO to have such low tracking record. We found that data on university destination were more likely to be missing for those who were White (550 or 63% of those not tracked as compared to 980 or 53% of those tracked) and with lower GCSES (450 or 51% of those not tracked had 0 to 3 GCSEs compared to 690 or 37% of those tracked). Conversely, those tracked are more likely to be Asian and also were not in receipt of discretionary payments. The lower attainment of the non‐tracked group would align with a hypothesis that these students have not entered higher education at all, giving the explanation for no tracked destination being found but the data themselves are inconclusive.

The information known about each participant on entry to the WP program was gender, ethnicity, if parents had higher education experience, their preferred choice of university course aged 16, their age 16 exam results and subjects, and if they received a bursary (a discretionary payment—allocated to students with a low household income). The POLAR data^21^ that divide areas of the United Kingdom into participation rates in higher education was known for the students. Two versions of POLAR data, 2 and 3, were used in the different years but they were analyzed together as considered broadly compatible. POLAR data were categorized into two groups, POLAR group 1, 2 (representing the lowest rates of higher education entry) and POLAR group 3, 4, and 5 (representing the highest rates of higher education entry) to enable descriptive analysis.

The preferred course at university was derived from RO's question to the participants on joining the scheme: “Have you already considered what subject you may want to study at university?” If more than one area was mentioned only the first one was retained unless it was medicine then this was retained.

All the nonnumerical information was categorized into groups called “values” and each value was assigned a numerical code. Missing data were coded as a separate value. The subject participants interested in studying were divided into six values.

Ethnicity was converted into five groups: White, Asian, Black, and other, and missing.

The grades achieved in the exams at age 16 were given a score that counted all the participants' A and A* grades (the top grades). Exams taken at age 18 were converted to a numerical number with *A* = 5 points, to *E* = 1 point, and their top three grades were only used.

The data were analyzed on IBM SPSS Statistics Data Editor V25. Descriptive statistics were generated to discover if there were any common characteristics among those that studied medicine. In addition, multivariate regression was carried out—standard assumptions of independence of predictors held. Data were not analyzed in years of entry to the scheme as the number of students who went on to study medicine in the whole population was low. Also, there was no hypothesis considering changes in medical aspirations over time and nothing in the data or policy context to expect annual variations.

### 2.3 Multivariate regression

In building the multivariate regression in SPSS, we expect from the bivariate association that the exams at age 16 are the strongest predictor of studying medicine and that advanced exams taken at age 18 might also play a role. In terms of social background, ethnicity, POLAR quintile, and discretionary payments are expected to be significant. However, when including background and academic factors simultaneously such background effects might disappear.

We based our regression analysis—predicting studying medicine at university—on the 1850 cases who matched to their university destination (69.3% of the original study) and who also matched a complete set of age 16 exam results resulting in 1840 cases for the analysis.

Each factor was individually entered to test significance as a univariate predictor of studying medicine in a binary logistic regression model with medicine at university coded as 1 (Table 1).

**TABLE 1:** Logistic regression analysis predicting enrolment in medical school using social background, attainment, and intention

	Univariate model			Full model without A‐levels		Full model with A‐levels	
Predictor (reference category in brackets)	Beta	SE	chi‐square	Beta	SE	Beta	SE
Constant				−8.20**	.67	−13.83**	1.68
Background							
Gender (female)	−.44	.31	2.05				
Ethnicity			21.0**				
Ethnicity Asian (comparator White)	1.41**	.34					
Ethnicity Black, other, missing (comparator White)	1.16*	.41					
POLAR			3.79				
POLAR 1,2 (comparator 3,4,5)	−.61	.36					
POLAR not known (comparator 3,4,5)	−.6	.33					
Parental HE experience (Yes)	.29	.53	.30				
Discretionary Payments (yes)	.69*	.3	5.7*				
Intent and Attainment							
Intention to Study Medicine (yes)	3.48**	.36	132.64**	3.08**	.37	3.76**	0.52
Eligibility for Medicine (Yes)	.54**	.17	12.94**				
Number of GCSEs at A or A*	.52**	.06	105.14**	.47**	.07	.28*	0.95
A‐level Score (*A* = 5, *B* = 4 etc)	.48**	.07	63.34**			.50**	0.1
Model statistics							
n				1841		1423	
df				2		3	
Model −2 Log‐likelihood				294.923		169.35	
Chi‐Square				199.56*		169.48*	

*Note*: **Signifies *P* value <.001; * signifies *P* value <.05.

## 3 RESULTS

Fifty‐five participants out of 1795 went on to study medicine (see Table 2). This is a surprisingly small number, but only 336 participants ever expressed an intention of wanting to study medicine.

**TABLE 2:** Social background in relation to medicine aspiration, eligibility, and enrolment. All numbers have been rounded to the nearest 5 as per HESA guidance

	Participants, n (%), bold signifies adjusted residuals significant at >1.9 or <−1.9					
Background	Intention to Study medicine (n = 2665, count [%])		Eligibility for medicine (n = 2665, count [%])		Studying medicine at university (n = 1850, count [%])	
	Yes	No	Yes	No	Yes	No
Gender						
Female	215 (64)	1545 (66)	**950 (61)**	**810 (73)**	40 (75)	1175 (66)
Male	120 (36)	785 (34)	**610(39)**	**300 (27)**	15 (25)	620(35)
Ethnicity						
Asian	**165 (49)**	**580 (25)**	**520 (34)**	**220 (20)**	**30 (55)**	**545(30)**
Black	**65 (19)**	**225 (10)**	**195(13)**	**95 (8)**	10 (16)	205 (11)
White	**90 (26)**	**1420 (61)**	**765 (49)**	**745 (67)**	**15 (24)**	**970 (54)**
Other	**20**	**70 (3)**	60 (4)	30 (3)	5 (6)	60 (3)
Not known	5	40 (2)	20	20	0	20
POLAR quintile						
1,2	**150 (44)**	**1180 (51)**	765 (49)	560 (51)	25 (42)	870(48)
3,4,5	**90 (26)**	**460 (20)**	330 (21)	215 (20)	**15**	**355 (20)**
Not known	105 (31)	680 (29)	455 (29)	330 (30)	15	570 (27)
Parental HE experience						
Yes	25 (8)	310 (6)	90 (6)	70 (6)	5 (7)	100 (6)
No	135 (92)	2195 (94)	1465 (94)	1040 (94)	50 (93)	1695 (94)
Discretionary Payments						
Yes	**230 (68)**	**1335 (58)**	920 (59)	650 (58)	40 (71)	985 (55)
No	**110 (32)**	**990 (43)**	635 (41)	460 (42)	**15 (29)**	**805 (45)**

The 55 participants who studied medicine were significantly more likely to be Asian, more likely to be living in an area with high university attendance, more likely to receive discretionary payments, and less likely to be White. Table 2 separates the participants into those eligible, intending, and actually studying medicine. It shows that the participants who were eligible to study medicine were statistically more likely to be male, Asian, and significantly less likely to be White. Asians and those receiving discretionary payment were overrepresented among those intending to study medicine, Whites, and those from the lowest higher education participation areas were underrepresented. Caution is applicable in interpreting these associations due to the small number of participants.

There was significant variation in the age 16 exam (GCSE) grades for different groups (Table 3). The number of GCSEs at the highest grades (A or A*) was significantly higher for those intending to study medicine, those eligible to study medicine, and those actually studying medicine compared with those with no intention of studying medicine, those who were not eligible, or those studying another subject. For example, those currently studying medicine had a mean of nine top grades, whereas those who were not studying medicine had a mean of 4.7. Advanced exam scores (A levels) were also higher in those that studied medicine. Asian and “Other” ethnicity participants in this study outperformed the White and Black participants in terms of highest age 16 exam grades. Those from the areas with the highest participation rates in higher education also outperformed those from the lower participation rate areas.

**TABLE 3:** The association between GCSE and A‐level attainment and social background, and medicine aspiration, eligibility, and enrolment

	Significant figures *P* < .05 in bold *T*‐test for binary variables and ANOVA for those with three or more categories (between‐group variance significance)					
	Number of A/A* at GCSE (max n = 2665)			Mean A‐level score (max n = 1424)		
	n	Mean	SD	n	Mean	SD
Intention to study medicine						
Yes	335	**6.2**	3.2	190	10.5	3.6
No	2305	**4.3**	3.0	1235	10.1	3.5
Eligibility for Medicine						
Yes	1550	**5.4**	3.1	863	10.3	3.5
No	1095	**3.5**	2.8	560	10.0	3.5
Studying Medicine						
Yes	55	**9.0**	1.9	35	**14.4**	1.7
No	1785	**4.7**	3.0	1390	**10.0**	3.5
Gender						
Female	1745	4.7	3.2	940	10.1	3.4
Male	895	4.4	2.9	485	10.2	3.6
Ethnicity						
Asian	740	**4.8**	3.1	415	10.0	3.7
Black	285	**4.2**	3.1	170	10.0	3.1
White	1500	**4.5**	3.1	780	10.3	3.5
Other	90	**5.0**	3.1	45	10.5	3.9
Not known	25	**3.4**	2.4	10	8.3	2.4
POLAR						
1,2	1315	**4.5**	3.1	628	10.0	3.6
3,4,5	540	**5.0**	3.1	240	10.0	3.4
Not known	785	**4.4**	3.1	555	10.4	3.4
Parental HE experience						
Yes	160	4.8	2.9	50	10.3	3.2
No	2485	4.6	3.1	1370	10.1	3.5
Discretionary Payments						
Yes	1550	4.7	3.1	640	10.2	3.4
No	1095	4.5	3.1	785	10.1	3.5

**FIGURE 1:**
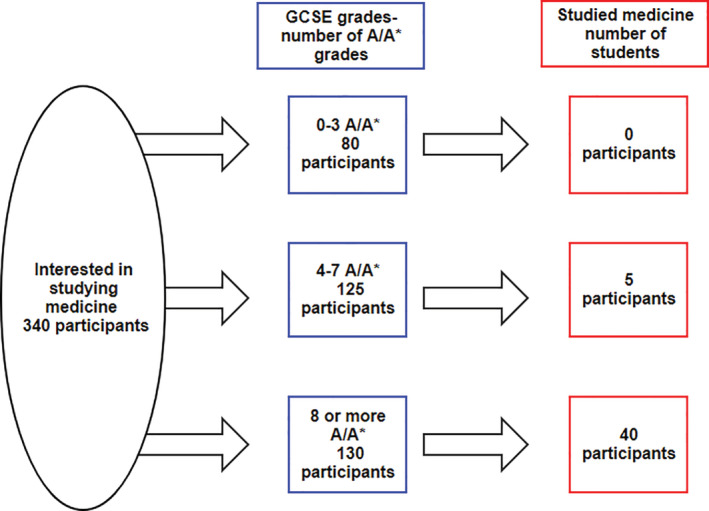
General Certificate in Secondary Education achievement and university destination of those participants who were interested in studying medicine

Given the wide‐reaching importance of exams at age 16 (GCSE results), Figure 1 describes the relationship between GCSEs and studying medicine in more detail. It illustrates that the majority of participants who were “interested in studying medicine” and who went on to study medicine had achieved eight or more top grades at GCSE (89%). No students studying medicine had achieved fewer than three top grades yet 80, a quarter (24%) of the participants who expressed an interest in studying medicine at age 16 had fewer than three grade GCSEs making them academically unlikely to be competitive for pursuing their interest in medicine.

### 3.1 Multivariate regression

For background factors, the chi‐squares varied from 0.3 for parental higher education to 21.0 for ethnicity. The most powerful univariate predictors were intention to study medicine (*χ* ^2^ = 132.6, *P* < .01); number of A and A*s (*χ* ^2^ = 105.1, *P* < .01), and A‐level score (*χ* ^2^ = 63.3, *P* < .01), although, perhaps surprising, eligibility for studying medicine was significant but added comparatively less explanatory power (*χ* ^2^ = 12.9, *P* < .01). The multivariate investigation showed that the most parsimonious model with the fewest predictors of studying medicine is a model of knowing GCSE scores and the intention to study medicine (*χ* ^2^ = 199.60, *P* < .01, 2 DF, n = 1841): Adding other factors and the associated degrees of freedom is not associated with an improvement to model fit, or significance. This is true for eligibility for medicine and A‐level scores, although A‐level scores are the only additional significant predictor after knowing GCSE scores, and intention to study medicine, although the number of unknowns regarding A‐level reduces the sample size, and thus model fit statistics (*χ* ^2^ = 169.48, *P* < .01, 3 DF, n = 1423). None of the social factors: ethnicity, gender, POLAR, parents having HE experience, and eligibility for discretionary payments are significant when added to the base model or increase the model fit. We tested an interaction effect between gender and GCSE scores and gender and intention for medicine, as men seemed underrepresented, neither main nor interaction term was significant.

## 4 DISCUSSION

Which disadvantaged students study medicine? In this study, these students were more likely to be Asian, and less likely to be White, they were living in an area with high university attendance and more likely to receive discretionary payments, were high attaining in exams, and had an expressed interest to study medicine from age 16.

The RO study participants were mostly from areas with low rates of higher education entry and all from underperforming state secondary schools but in line with the national average, 3% went on to study medicine.^22^ This is noteworthy as medical students in the United Kingdom are often privileged,^23^ with 50% to 70% of parents having experienced higher education, and 45% from the highest university participation neighborhoods, and at least 20% from fee‐paying schools. From the data we were unable to judge whether this success is due to RO,^17^ or due to the RO students being a more motivated group, having chosen to participate in RO^13^ or another unknown factor.

More females than males entered medical school despite equal numbers being interested in studying medicine and similarly high exam grades at age 18. University statistics for students entering medical school in 2017 found the female‐to‐male ratio 1.4:1 and the present study 2.6:1.^24^ White males from the lower socioeconomic households underachieve in education in the United Kingdom.^25^ The RO males are academically successful but this is not translating into medical school places. This study is not able to determine whether male participants were applying to medicine but not receiving offers, or ultimately deciding to apply for different degrees and careers. Research in America found females can be more successful at multi‐mini application interviews^26^ used in many medical school applications and similar findings were seen in a Scottish study.^27^ To establish that the males in this study are failing at the application stage would require further research. However, it is encouraging that the WP male students are achieving academically, which might suggest that more motivated male students joined the RO scheme or that it may have motivated or contributed to their success.

The concern from this database is the lack of White students who were interested in medicine—they represented 57% of the population but they accounted for only 19% of the population interested in studying medicine in contrast to the Asian students of whom represented 28% of the population but almost a half of the students wanted to pursue medicine as a career. It has been found that Asian parents emphasize the duty that students have to succeed and this success is limited to a narrow field of options that include medicine.^28^ The pressure placed on these students would not be an appropriate strategy to apply to all students but having high aspirations for all students expressed by teachers, career advisors, and WP organizations would be appropriate.

These high aspirations would need to be appropriate to the individual students as some students in this study appeared to have unrealistic aspirations when considering medicine as a career at age 16. The UK medical schools vary on the weighting they place on exams at age 16. Most medical schools specify five GCSEs with a minimum of C in English and Maths, but some require six GCSE passes at A or above to include English, Mathematics, and two Science subjects.^29^ This study found most students (89%) who obtained a medical school place had more than eight top grades (A/A* grades) at GCSE, and 24% of students who joined the scheme expressing an interest in studying medicine had less than 3. There were 550 students (20%) who received more than eight top grades (A/A*) at GCSE, and 80 (14%) of these students studied subjects allied to medicine. This is a pool of widening participation of students who appear to have the academic credentials for eligibility for medical school. A way to tackle the persisting socioeconomic inequalities in access to medical school could be to target the academically able students, as identified by their exam grades at age 16. They could be encouraged to consider medicine as a realistic career for them, given help with medical school applications, and investment made into ensuring that they achieve top exam grades.

### 4.1 Strengths and limitations of the study

The strength of this longitudinal study was the ability to track students from joining a WP scheme, to their entry to university via HEAT data, and that the key findings resonate with previous research. RO ensured that participants were only eligible to join the scheme if they fulfilled two or more WP criteria, and they only accepted participants from targeted state schools. A unique feature of this study was the recording, at age 16, of the subject that participants were interested in studying at university. This enabled correlations to be made between this interest and the subject they studied at university. Data were also used from five cohorts of participants increasing the sample size and strengthening the reliability of the data. However, as with all research, there were some limitations. The data were not collected specifically for this study and this may have affected the accuracy of the recorded data needed for this research question. The destination of the students who did not have a university destination recorded was not recorded; however, if a university was not matched using the HEAT data, it is likely that they did not enter university immediately after school. This may be a heterogeneous group, comprising participants who did not go to university, those who studied abroad, and those who took time out before going to university, and those for whom the linkage procedure failed. The exam grades were converted into a numeric score without reference to the subjects studied. Some medical schools stipulate which subjects students study for their exams at age 18. Therefore, students might have a high numerical score but with qualifications in the wrong subjects for medicine. Furthermore, a range of factors influence studying medicine, including parental and community attitudes and individual personalities and values. Our study is silent on these factors. There were missing data in all the variables, which should be considered when assessing the outcomes.

## 5 CONCLUSION

The 2018 report by the Medical Schools Councils^3^ reports that the national demographic data are showing there has been progress made in ensuring that there is equality in admission to medical school and that there is now increasing ethnic diversity. However, there is still concern that little progress has been made for those with social and educational disadvantage and this is where the concentration of effort is needed. If 8 or more top exam grades at age 16 are most associated with gaining a medical school place, then this information is valuable for those delivering career advice to students, for those devising WP schemes, and for medical schools. It also highlights that attainment is a key barrier for access, and that more work is required to soften the association between social background and attainment. This study identified a group of students who were considering medicine as a career but did not have the exam grades at age 16 sufficient for success. This group would benefit from input to maximize their attainment and to explore other career opportunities.

The reasons for the low proportion of WP men entering medicine require further research, to see if barriers can be overcome. If the barrier to success is the medical school interview or the application process, then career advisers and widening participation schemes could target resources in these areas. If the barrier is their perceptions of the career itself then this could be a priority for focus in a widening participation program or through the media. In addition, understanding the reasons for some academically able WP student choosing to go to university but not to study medicine would provide valuable insights into factors influencing student's career choices.

Further evaluation is required to understand which, of the multiple strands of the Realising Opportunities WP Scheme, might help participants to successfully apply to medical school, or whether it is the combination of the strands that is beneficial. To increase the WP proportion in medicine, further qualitative research is needed to understand if there are surmountable barriers preventing this group from considering medicine as a career. If all these barriers can be successfully targeted then a more diverse medical school population could develop.

## CONFLICT OF INTEREST

The authors declare no conflicts of interest regarding this study.

## AUTHOR CONTRIBUTIONS

Conceptualization: Carolyn Murray, Anna Mountford‐Zimdars, Karen Mattick

Data Curation: Carolyn Murray

Formal Analysis: Carolyn Murray, Anna Mountford‐Zimdars

Funding Acquisition: Not applicable

Investigation: Carolyn Murray, Anna Mountford‐Zimdars

Methodology: Carolyn Murray, Anna Mountford‐Zimdars, Karen Mattick

Project Administration: Carolyn Murray

Resources: Carolyn Murray

Software: Carolyn Murray, Anna Mountford‐Zimdars

Supervision: Anna Mountford‐Zimdars, Karen Mattick

Validation: Carolyn Murray, Anna Mountford‐Zimdars

Visualization: Not applicable

Writing—Original Draft Preparation: Carolyn Murray

Writing—Review and Editing: Carolyn Murray, Anna Mountford‐Zimdars, Karen Mattick

  All authors have read and approved the final version of the manuscript.

  Carolyn Murray confirms that she had full access to all of the data in this study and takes complete responsibility for the integrity of the data and the accuracy of the data analysis.

## TRANSPARENCY STATEMENT

Carolyn Murray affirms that this manuscript is an honest, accurate, and transparent account of the study being reported; that no important aspects of the study have been omitted; and that any discrepancies from the study as planned have been explained.

## Data Availability

The data that support the findings of this study are available from the corresponding author upon reasonable request.
